# Climate change, future Arctic Sea ice, and the competitiveness of European Arctic offshore oil and gas production on world markets

**DOI:** 10.1007/s13280-017-0957-z

**Published:** 2017-10-24

**Authors:** Sebastian Petrick, Kathrin Riemann-Campe, Sven Hoog, Christian Growitsch, Hannah Schwind, Rüdiger Gerdes, Katrin Rehdanz

**Affiliations:** 1 Kiel Institute for the World Economy, Kiellinie 66, 24105 Kiel, Germany; 2 Alfred-Wegener-Institut Helmholtz-Zentrum für Polar- und Meeresforschung, Bussestrasse 24, 27570 Bremerhaven, Germany; 3 IMPaC Offshore Engineering, Hohe Bleichen 5, 20354 Hamburg, Germany; 4 Center for Economics of Materials, Fraunhofer IMWS, Walter-Hülse-Str. 1, 06120 Halle, Germany; 5 Department of Economics, Kiel University, Olshausenstrasse 40, 24098 Kiel, Germany; 6 Institute of Energy Economics at the University of Cologne, Alte Wagenfabrik, Vogelsanger Str. 321a, 50827 Cologne, Germany

**Keywords:** Arctic climate change, Arctic sea ice, Offshore oil and gas production, Oil and gas prices

## Abstract

A significant share of the world’s undiscovered oil and natural gas resources are assumed to lie under the seabed of the Arctic Ocean. Up until now, the exploitation of the resources especially under the European Arctic has largely been prevented by the challenges posed by sea ice coverage, harsh weather conditions, darkness, remoteness of the fields, and lack of infrastructure. Gradual warming has, however, improved the accessibility of the Arctic Ocean. We show for the most resource-abundant European Arctic Seas whether and how a climate induced reduction in sea ice might impact future accessibility of offshore natural gas and crude oil resources. Based on this analysis we show for a number of illustrative but representative locations which technology options exist based on a cost-minimization assessment. We find that under current hydrocarbon prices, oil and gas from the European offshore Arctic is not competitive on world markets.

**Electronic supplementary material:**

The online version of this article (doi:10.1007/s13280-017-0957-z) contains supplementary material, which is available to authorized users.

## Introduction

A significant share of the world’s undiscovered oil and natural gas resources is assumed to lie under the seabed of the Arctic Ocean. The US Geological Survey estimates that “about 30% of the world’s undiscovered gas and 13% of the world’s undiscovered oil” are north of the Arctic Circle, including onshore resources (Gautier et al. 2009). Up until now, the exploitation of the resources especially under the European Arctic has largely been prevented by the challenges posed by temporary sea ice coverage, harsh weather conditions, darkness, remoteness of the fields, and lack of infrastructure such as Search and Rescue (SAR) facilities. The need for special equipment, suitable for winter operation, including ships and platforms, and the long distance to existing infrastructure make exploration and production activities in the Arctic Ocean especially costly compared to other, even non-conventional sources of hydrocarbons. Consequently, competition by less expensive supply options has led producers to leave European Arctic resources so far largely untapped.

Gradual warming has, however, improved the accessibility of the Arctic Ocean and raised hopes among hydrocarbon producers that envisage a diversification of their portfolios away from less politically stable or depleting sources elsewhere. Also, oil and gas importers in Europe are interested in reduced dependence on traditional suppliers in Russia, the Middle East, or Africa that are perceived as geopolitically risky. At the same time, environmentalists see the pristine Arctic ecosystems in danger of pollution by oil and gas production facilities and associated infrastructure. Also, the long-term need of fossil fuels given the Paris Agreement and international climate protection goals question the rationale of exploiting Arctic fuel resources. The implications of additional production of energy resources from the European Arctic on the environment, energy markets, and geopolitics warrants a closer look on whether, where, and under which conditions additional Arctic offshore oil and gas production is desirable.

We show for the most resource-abundant European Arctic Seas whether and how a climate change-induced reduction in sea ice might impact future accessibility of offshore natural gas and crude oil resources. Based on this analysis we show for a number of illustrative but representative locations which technology options are likely based on a cost-minimization assessment. We contribute to the literature by combining geology-based assessments with climate model-based projections on sea ice development, engineering-based technology assessments, and information on global oil and gas prices to assess economic viability of offshore oil and gas production in the European Arctic.

Existing studies stem mostly from the gray literature acknowledging that the European part of the Arctic Ocean has a significant resource potential that could play a significant role on the global hydrocarbon markets, but come to mixed conclusions regarding the economic viability of Arctic oil and gas from European offshore sources (IEA 2013; Aarhus et al. 2014; Casey 2014; Emmerson and Lahn 2012). Here, we focus mainly on studies published after the drop in oil and gas prices during the 2007/2008 financial crisis. From a conceptual point of view, our work relates to Harsem et al. (2015), who studied the impact of projected sea ice developments on oil activity in 21 Arctic oil provinces. They conclude that (Harsem et al. 2015, p. 101), “while certain Arctic provinces may become more attractive during the next 30 years, most provinces will still imply very high costs. Even though the Arctic is expected to lose a significant portion of its sea ice cover, this does not automatically imply that it will become a substantial oil region.” Their work, as ours, is based on USGS estimates for oil potential, but applies only one climate model and a top-down approach for estimating cost differentials between the provinces instead of bottom-up estimates of cost levels. Aarhus et al. (2014), in a report prepared by the Norwegian gas pipeline operator with input from the Barents Sea Gas Infrastructure Forum (BSGI), reviewed both potential technologies and potential production costs. They concede that, while acknowledging the potential of the Barents Sea, existing discoveries are not sufficient to justify investment in new gas infrastructure, which will be challenging to realize from a post-tax project robustness perspective. Casey (2014) focused on licensing rounds in Greenland and was skeptical about economic feasibility, which, if at all, he only saw in the long term. The IEA (2013, p. 138) concludes that exploration and production activity in the offshore Arctic is likely to increase in the coming decades, but qualifies that “realising the potential of Arctic resources will depend not only on market success and conditions in finding them, but also on technological innovation and the ability to exploit these resources in a safe and environmentally sound manner. The cost of bringing Arctic resources to markets is substantial, so projects will require a high market price and more cost-effective technology to attract investment.” Lindholt and Glomsrod (2012) found in their scenario analysis on the entire Arctic that its relevance for international gas markets will decline and for oil markets at least not increase. Emmerson and Lahn (2012) highlighted the importance of fluctuations in energy prices for energy developments in the Arctic and show cost estimates of 30–100 2008 USD/barrel of oil, based on IEA estimates. In the same vein, Overland et al. (2015) showed that oil and gas production depends crucially on price development, the level of international cooperation, and climate policy.

In the course of the paper we will first describe estimates of oil and gas potential in the European offshore Arctic, then analyze results from global climate models to draw conclusions on Arctic sea ice development in 2040 under two climate scenarios. We then present results from a bottom-up, engineering-based cost estimation exercise for a wide variety of technologies for offshore oil and gas production. Finally, we combine these results to estimate likely production costs in various exemplary Arctic regions. We conclude by showing that under current hydrocarbon prices, oil and gas from the European offshore Arctic are not competitive on world markets. At the same time, the impact of a changing accessibility of the Arctic Ocean under climate change on the operability of production technologies as well as economics of production is only minor.

## Materials and Methods

### Estimated oil and gas volumes in the European Arctic

**Fig. 1:**
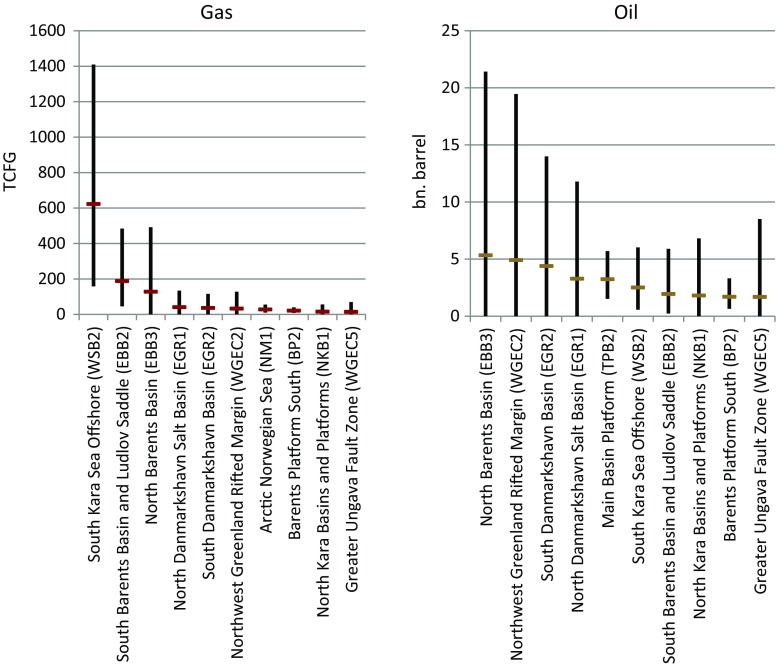
Geological assessment units in the European Arctic with the highest gas (in trillion cubic feed of gas, TCFG) and oil (in billion barrel) potential. Horizontal lines (red for gas and brown for oil) show mean estimated volumes, vertical bars (in black) show the minimal volumes that are associated with the assessment units with a 5% (upper bound) and 95% (lower bound) chance. Source: Own presentation based on USGS (2008b)

**Fig. 2:**
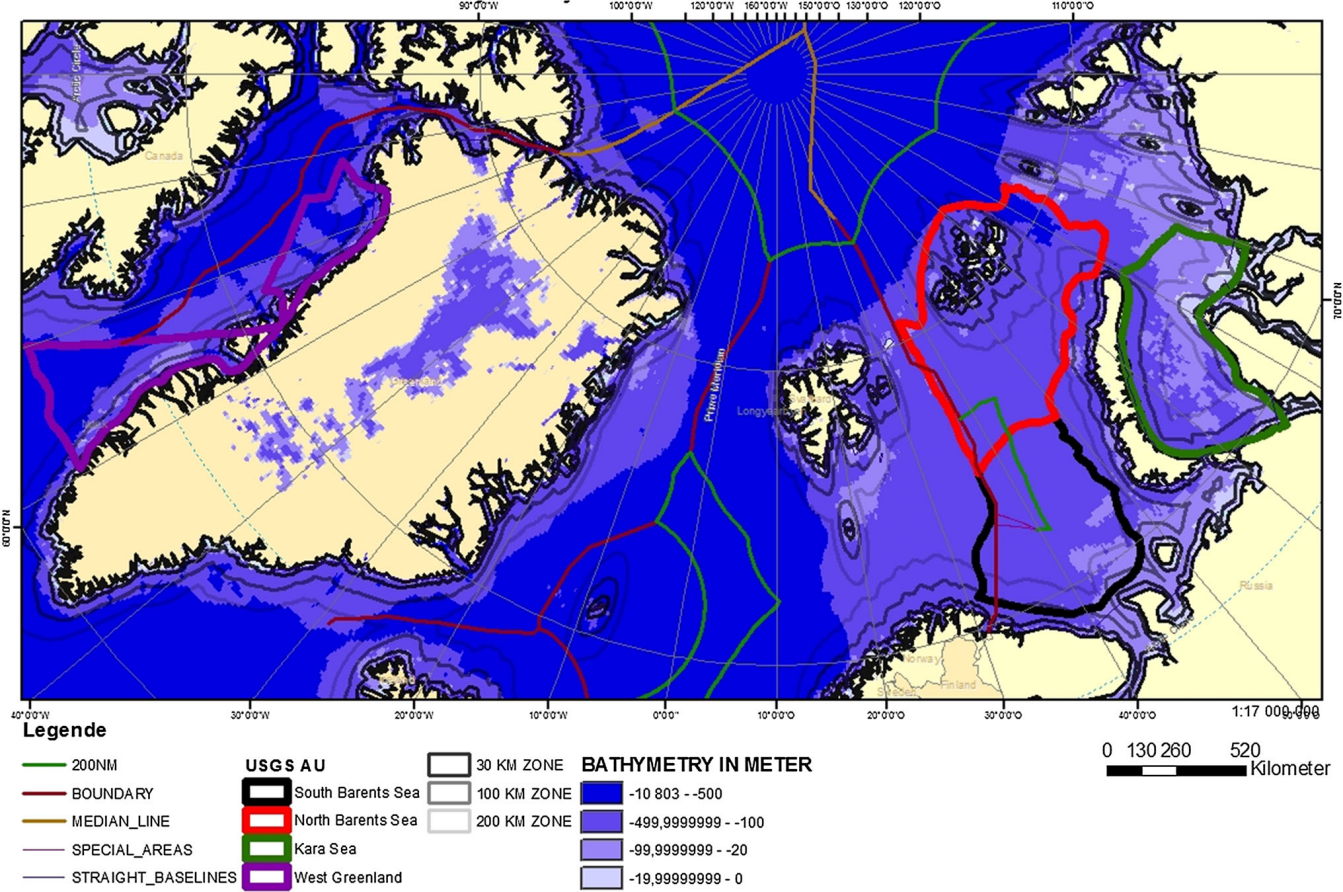
The four assessment units including step-out distance and bathymetry. International borders are from IBRU (2012). Source: Own presentation based on USGS (2008b)

The more favorable the pre-drilling conditions are in terms of the size of fields, the distance to existing infrastructure, bathymetry, as well as weather and ice conditions, the more likely are exploration drilling campaigns in this area and, consequently, later production of hydrocarbons. Today, the USGS’ Circum-Arctic Resource Appraisal (USGS-CARA 2008a; Gautier et al. 2009) provides the most recent information on potential volumes for predefined assessment unit. Figure 1 shows estimated volumes from the USGS-CARA for the ten largest European assessment units north of the Arctic Circle with regard to potential gas or oil volumes. To account for the size of assessment units in the European Arctic with the highest gas and oil potential, but also for differences in bathymetry and future ice conditions, our analysis focuses on the following four assessment units: the “South Kara Sea” (WSB2), the “South Barents Basin and Ludlov Saddle” (EBB2), the “North Barents Basin” (EBB3), and the “Northwest Greenland Rifted Margin” (WGEC2).^1^ The four assessment units also represent a wide coverage of jurisdictions (Fig. 2). Both the Kara Sea and parts of the Barents Sea areas are in the Russian continental shelf, the larger part of the Barents Sea is in the Norwegian EEZ (including the continental shelf) and the West Greenland assessment unit is under Danish, or rather Greenland’s jurisdiction. While fields in the southern Barents Sea are already under exploitation and infrastructure is already developed, the other three assessment units are more distant from e.g., port infrastructure and generally less developed.

### Future Arctic Sea ice distribution

It is impossible to predict the Arctic sea ice distribution for the coming decades. However, global climate models are able to estimate likely sea ice distributions under assumed emission scenarios. Global coupled models are used in standardized experiments, known as the Coupled Model Intercomparison Project phase 5 (CMIP5), to project possible climate change magnitudes until 2100 (Taylor et al. 2012). We compare 34 of these CMIP5 models (see Table S1) with satellite-derived observations to select four CMIP5 models, which compute sea ice concentration best compared to these observations. The selected models are further analyzed to estimate the future sea ice distribution in our four target areas. We focus on results from the emission scenarios RCP 4.5 and RCP 8.5 (Moss et al. 2010), named after the Representative Concentration Pathways adopted by the IPCCs fifth assessment report and representing different developments in greenhouse gas concentrations and radiative forcing, respectively.

**Fig. 3:**
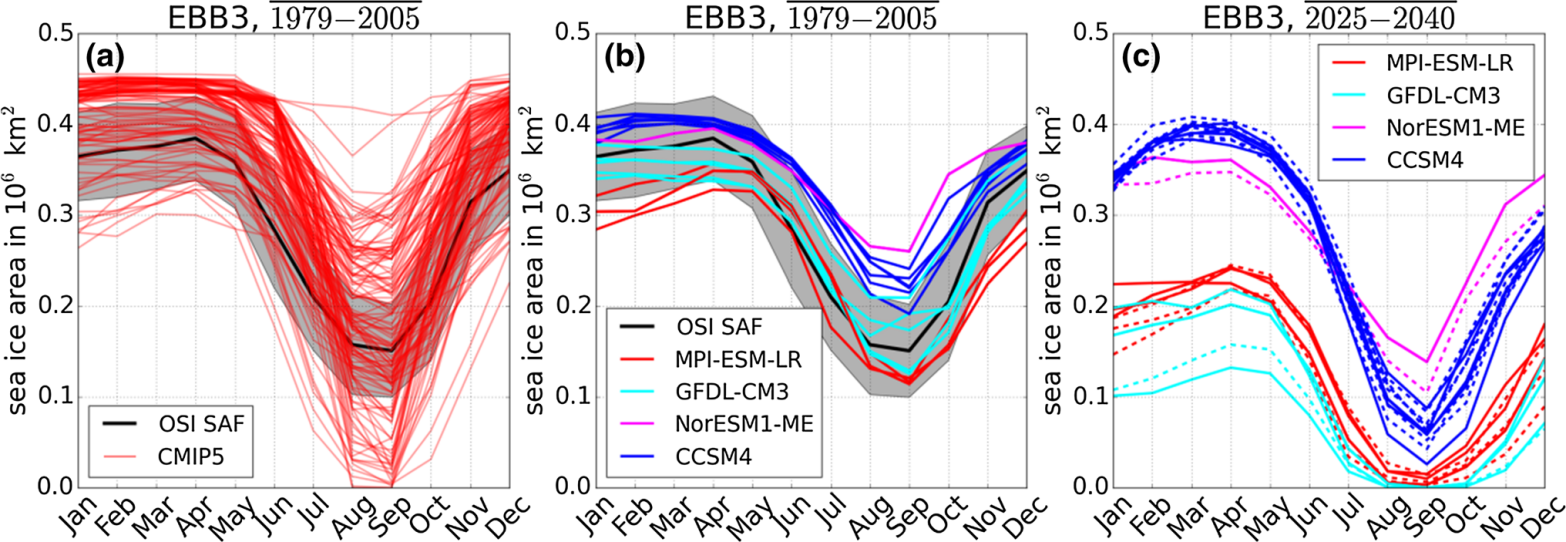
Mean seasonal cycle of sea ice area in million km^2^ for the region EBB3 in the northern Barents Sea. **a** Mean over the years 1979–2005 of satellite-derived data OSI SAF (mean: solid line, standard deviation: gray shading) and single ensemble members of CMIP5 models; historical simulation. **b** Mean over the years 1979–2005 of satellite-derived data OSI SAF (mean: solid line, standard deviation: gray shading) and four selected CMIP5 models; historical simulation. **c** Mean over the time period 2025–2040 from for CMIP5 models with emission scenarios RCP 4.5 (solid lines) and RCP 8.5 (dashed lines)

The seasonal cycle of sea ice area covering the northern Barents Sea, target area EBB3, is shown as an example in Fig. 3. Results from other target areas are shown in Figures S1 and S2. Different realizations of the same model experiment are called ensemble members. All ensemble members of 34 CMIP5 models are compared to the sea ice concentration product OSISAF^2^ in Fig. 3a for the period 1979–2005. The very large range of simulated past sea ice areas in the CMIP5 models reveals that not all models are suitable for our analysis. Most models overestimate the sea ice area as well as the amplitude of the seasonal cycle. Therefore, we select four CMIP5 models on the basis of the model observation misfit from sea ice concentration in our target areas as well as in the entire Arctic. We compare the mean seasonal cycle for two time periods 1979–2005 and 1992–2005 of each ensemble member with OSI SAF and a second sea ice concentration product, SSM/I.^3^ The detailed comparison is described in Riemann-Campe et al. (2014).

Those four models with the lowest misfit in the target areas as well as in the entire Arctic are CCSM4, GFDL-CM3, MPI-ESM-LR, and NorESM1-ME (Fig. 3b). Note, that the number of ensemble members ranges from one (NorESM1-ME) to six (CCSM4) for the historical simulation of the models. The number of ensemble members varies also with the experimental design: historical, RCP 4.5 and RCP 8.5. Multiple ensemble members are needed to reveal the natural variability; the internal, unforced variability that occurs in nature as in model simulations for complex systems. This variability is relatively small in the historical simulation between 1979 and 2005 (Fig. 3b) with largest values of up to 0.1 million km^2^ in the GFDL-CM3. This variability increases for the CCSM4 and the GFDL-CM3 in the scenario simulation RCP 4.5 and RCP 8.5 with the largest variability of about 3.5 million km^2^ in the CCSM4 RCP 4.5. Note that the GFDL-CM3 has only one ensemble member for the RCP 8.5 simulation, thus determination of internal variability is uncertain.

Overall, the inter-model variability ranges over approximately 0.1 million km^2^ in March and 0.15 million km^2^ in September for the period 1979–2005 (Fig. 3b). This variability increases to 0.25 million km^2^ in March for the period 2025–2040 (Fig. 3c). Although the models simulate similar sea ice distributions for recent decades, their simulations of the September ice-covered area differ substantially for the coming decades. The variability induced by the emission scenarios RCP 4.5 and RCP 8.5 is small in comparison with inter- and intra-model variability. Its magnitude is about 0.05 million km^2^ until 2040. Much larger differences occur in the following decades after 2040.

Turning to the change of future seasonal cycle, the magnitude of the sea ice area seasonal cycle in all four models is similar during the period 1979–2005. In contrast, the magnitude of the seasonal cycle differs in the models for the period 2025–2040 (Fig. 3c). The NorESM1-ME and the CCSM4 show an increase in magnitude of the seasonal cycle for the target area EBB3: the maximum sea ice area is similar for the past and the future time period in March, while the minimum sea ice area decreases by 0.1–0.2 million km^2^ in September for the future time period. The ice melts faster in spring indicated by steeper gradients. This occurs in contrast to the gradient of the ice area increase during autumn and winter, which remains similar in both time periods. The freezing process takes longer before the maximum ice area is reached in March.

**Fig. 4:**
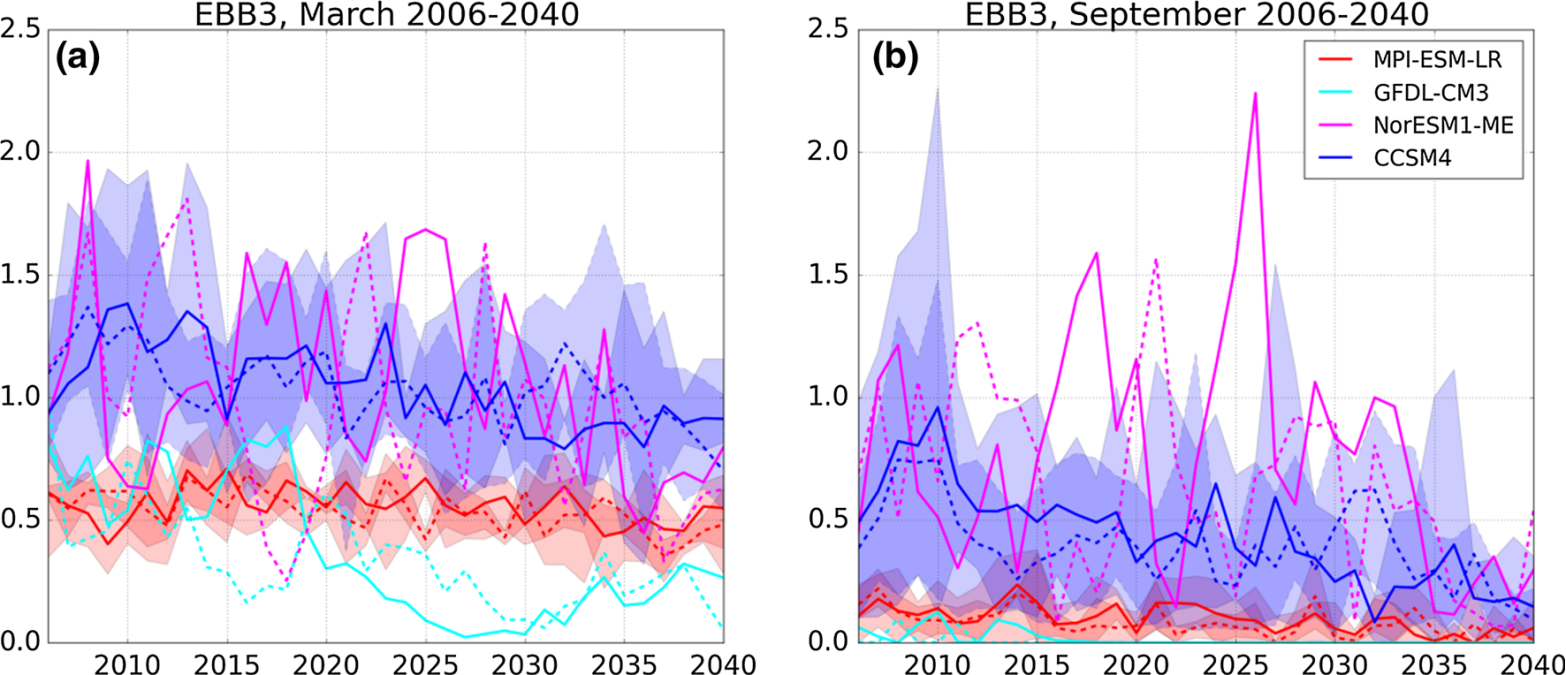
March (a) and September (b) mean sea ice thickness in m for the region EBB3 from four CMIP5 models with the emission scenarios RCP 4.5 (solid lines) and RCP 8.5 (dashed lines). Note: If more than one ensemble member is available per model, the mean is shown by the line and the range over all ensemble members per model is indicated by the shading. Overall, the four chosen models agree on a reduction of September sea ice area until 2040. However, not all models project a reduction of March sea ice area. All four models show a large annual variability. For sea ice thickness the annual variability is larger than the long-term reduction until 2040

Finally, the annual variability of sea ice thickness is shown in Fig. 4 for the summer minimum in September and the winter maximum in March. Note, that the CCSM4 and the NorESM1-ME simulate thicker ice in September than in March in 2010 and 2026, respectively. These September ice thickness maxima of about 2.2 m are based on local thick ice flows southeast of Franz Josef Land (CCSM4) and northwest of Novaya Zemlya (NorESM1-ME). Greater thickness in late summer compared to the end of winter indicates severe shortcomings of the models, most likely due to spatial resolution and the representation of sea ice dynamics. The annual variabilities of the MPI-ESM-LR and the GFDL-CM3 are much smaller than those of the CCSM4 and the NorESM1-ME. However, the magnitude of the annual variability in all models is larger than their long-term decrease until 2040.

### Technology portfolio and cost estimates for oil and gas production in the Arctic Ocean

To assess the economic viability we start by applying a bottom-up cost estimation exercise to estimate the cost associated with the different production technologies. Our cost estimates reflect an ex-ante assessment from an engineering perspective. In that sense, our cost estimates do not take into account any unforeseen ex-post developments. We estimate individually the cost contributions for the individual building blocks of the production technologies: fixed production facilities, shallow water production facilities, floating production facilities, and subsea production facilities.^4^

Fixed platforms are used where the water depth does not exceed roughly 250 m and where especially harsh environmental conditions like large waves, strong winds and currents, and drifting ice or icebergs are present. A number of production facilities in the Arctic are installed in shallow or very shallow waters with water depths ranging from 5 to 20 m. When concrete platforms are used these often provide large storage capacity in the hull structure allowing to temporarily store and export the product via shuttle tankers (discontinuous export). Alternatively and in case of water depth restriction, export of the products can also be realized by means of pipelines to backup treatment plants and further to the local network (continuous export). Such continuous pipeline connection could be used to produce LNG (Liquefied Natural Gas), CNG (Compressed Natural Gas), or GTL (Gas to Liquids) in a specialized treatment plant, allowing further long distance exports to clients worldwide.

Floating platforms can be designed to work in even harsher environments and especially in deep water conditions. An example is the FPSO (Floating Production, Storage, and Offloading) technology, where a floating terminal vessel (ship shaped or barge type) comprises capacity for production, processing, and storing of hydrocarbons. The product can be exported either via shuttle tanker (mostly if the step-out distance exceeds roughly 200 km) or via pipeline, depending on cost effectiveness. All floating production units are permanently moored to the seafloor. Most units can be temporarily disconnected from the moorings in emergency, e.g., when large icebergs or excessive thick drifting ice approaches, which cannot be managed by ice-breaking service vessels.

The latest development is the subsea processing technology where no permanently floating or fixed platform is required for production. Instead the production facilities are directly installed on the seafloor so that the environment at the sea surface does not harm the operations. These facilities export the products via pipeline to shore (so called subsea to beach (S2B) configuration) or via flowlines and risers to floating storage or production and storage facilities, such as FSOs (Floating Storage and Offloading facilities), FPSOs, or FLNG (Floating Liquefied Natural Gas (Production, Storage and Offloading)). It should be mentioned that each production unit mainly dedicated to gas production (or to oil production) will also produce condensate (comparable to oil) or associated gas and vice versa, which also has to be handled, or even stored and exported at the floating facility.

**Fig. 5:**
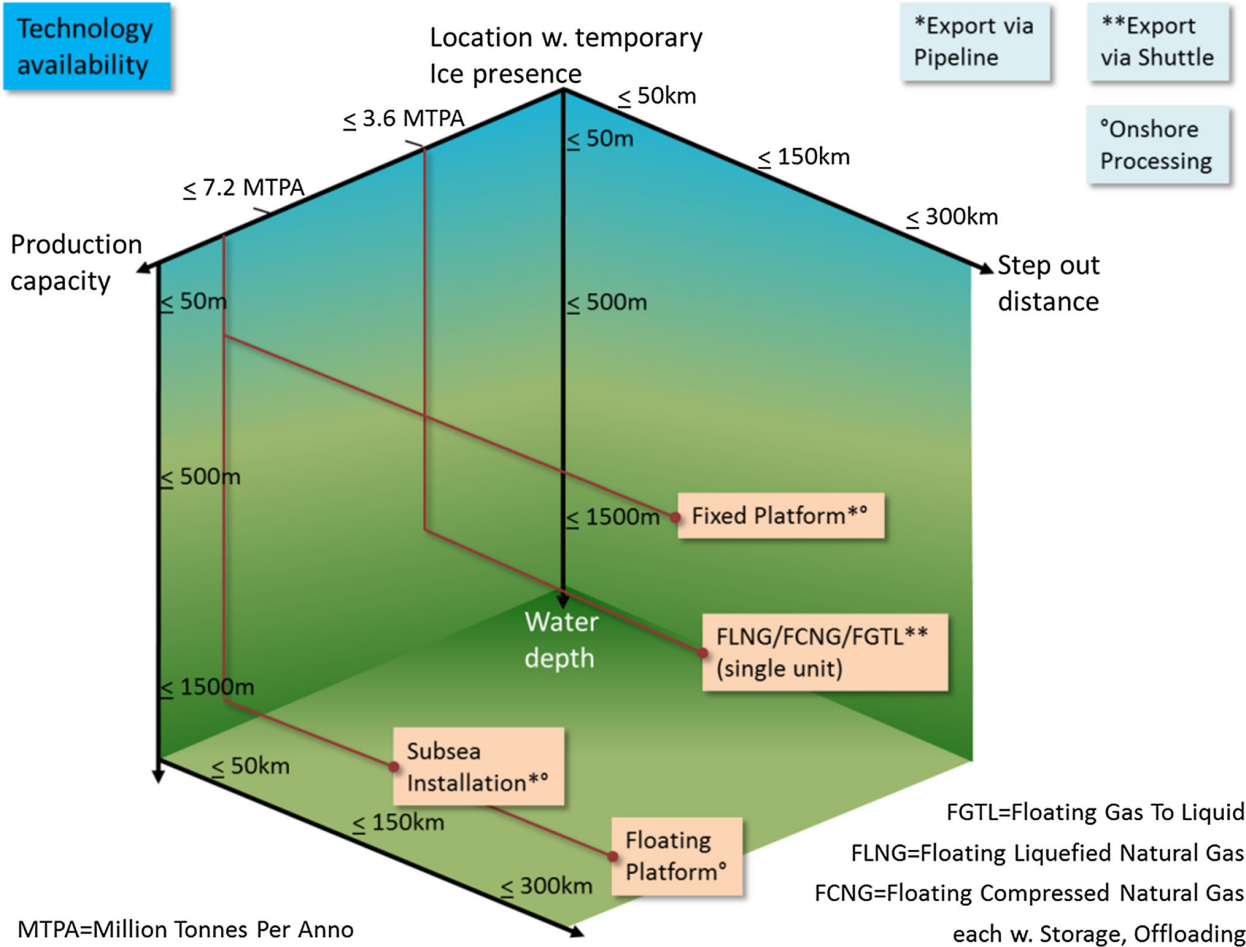
Suitability of available technology modules for oil and gas extraction in the Arctic. Source: Own presentation

The choice of the actual production technology employed at a given location depends on a number of environmental constraints, most importantly step-out distance, water depth, wave conditions (height, spectra, periods, and current), ice condition (type, thickness, extent, icebergs, ridges, drifting speed), and wind conditions (spectra, speed, gust). Assuming iceberg management for all technologies, Fig. 5 shows the available technology for offshore oil and gas extraction in the Arctic depending on field size, water depth, and distance to shore. A FLNG floating platform, for example, is best moored in up to 1500 m water depth, installed in a step-out distance to shore exceeding 300 km and designed with LNG production capacity of up to e.g., 3.6 MTPA.

It is important to note that deepwater areas, which are permanently ice covered, are very complicated (or even today not possible) to develop. This is caused by the need to get (vertical) access to the production wells for planned and emergency workover drilling and maintenance works by means of dedicated vessels (ships or semi-submersibles) and remotely operated vehicles (ROV). Directional drilling from aside (from onshore) is not feasible when the distance to the well exceeds about 8 km (4.32 nautical miles). Table 1 gives a summary of the feasibility assumptions.

**Table 1:** Feasibility assumptions of selected oil or gas production technologies

	Step-out distance (km)	Water depth (m)	Thickness of temporary sea ice (m)
Floating	≥ 200	≤ 500	≤ 1.5
Subsea	≥ 100	≥ 100	≤ 1.5
Fixed concrete platform	≤ 100	≤ 100	≤ 1.5
Shallow water production	≤ 30	≤ 20	≤ 1

Source: Own presentation. Iceberg management required for all technologies

Further constraints for operations at remote locations are e.g., set by rescue and evacuation systems and helicopter flights for personnel transfer. An installation is considered remote if it is located more than 40 nautical miles from the nearest manned installation or airport/heliport. Consequently, consideration should be given to provide Jet-A1 refueling facilities, where the distance from shore to an installation or vessel (with an operational helideck) exceeds 50 nautical miles.

In the following, we present the main cost components of the various technologies, including all project development costs, equipment, procedures, and operations needed to meet the specific requirements. The cost estimates are based on realistic technical and economic assumptions and cost data derived from comprehensive literature and internet source reviews (see Table S3 for more details). Note that the calculations have been carried out in the years 2011–2013. Meanwhile prices might have changed and technology further developed. Also, political constraints have changed (partly completely) in major Arctic areas since then. Therefore, we decided not to focus on this issue in greater detail.

All cost estimates include (if applicable) iceberg management by ice-breaking OSVs, project development, shore base, supply and tug boats, development drilling and completion, subsea components and installation, flowlines to gathering point, service vessels, structure decommissioning and removal as well as production, service, maintenance, and insurance. Shipping and onshore processing (if applicable) is included, too.

However, it is evident that the cost elements can only provide a rough estimate of the total costs. We do, for example, not include political costs, which may be required by the concessionaires of the deposits in the Arctic region. Political costs often include e.g., direct costs related to the permission award or to the provision of safety and security measures required by most of the involved communities or states. These political costs may drastically change the economic viability of an Arctic hydrocarbon export scheme up to conditions, where political costs have to be considered as a “show stopper.”

Table 2 shows our cost estimates for oil and gas extraction for the various technology options described above differentiating by operational expenditures (OPEX) and capital expenditures (CAPEX).

## Results

We combine the information from the suitability of a technology and the sea ice projections with data on bathymetry and step-out distance. Figure 2 above shows the step-out distance and bathymetry for the assessment areas in question, as well as international boundaries taken from IBRU (2012). The threshold values for step-out distance and bathymetry correspond to those in Table 1. Since most of the assessment units in question include landmass, each in principle comprises possible locations that allow for all technologies in question. Since we are interested in offshore oil and gas production, we do not consider shallow water production. Deep water prohibits the use of fixed platforms in the North and South Barents Sea (EBB2 and EBB3). Subsea and floating production technology remains possible in any assessment areas.

**Fig. 6:**
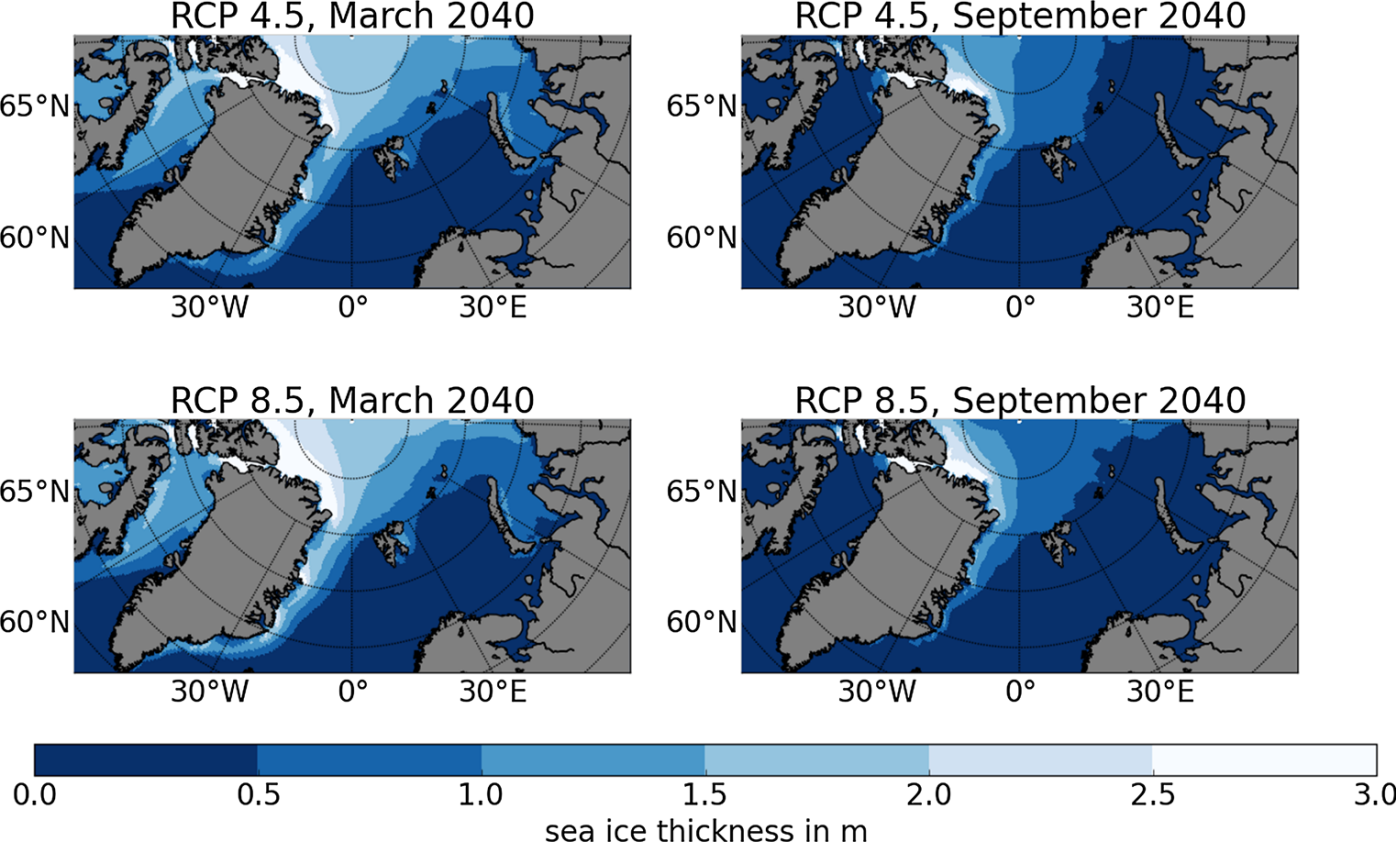
Multi-model summer and winter sea ice thickness under RCP 8.5 and RCP 4.5 (multi-model average of monthly maxima). Own presentation. Only MPI-ESM-LR, GFDL-CM3, NorESM1-ME were used for the analysis, since only these were available in sufficient timely resolution

Regarding ice conditions, we have to distinguish between summer and winter ice conditions. Fixed and subsea technologies can generally cope with harsh conditions and can be employed if ice conditions are favorable only in summer, where a time window with manageable ice conditions (1.5 m ice thickness or less) is needed for initial construction and maintenance. Floating production units as well as LNG carriers and tankers, however, need year-round sufficiently low ice thickness under 1.5 m. Figure 6 shows the average projected summer ice thickness in 2040 in the assessment areas under study, highlighting the 1.5 m thickness border for the climate scenarios with low (upper panel) and high (lower panel) radiative forcing. We concentrate on 2040 in order to get an idea of the long-run prospect and in order to get an idea about the potential impact of climate policy and climate change on the prospects for energy production in the Arctic Ocean. Even though the differences in ice thickness in the higher Arctic Ocean are visible, we can conclude that even in the scenario with relatively little climate change (RCP 4.5) offshore oil or gas production in the European Arctic Ocean in all assessment areas in question is not substantially hindered by summer sea ice.

While ice coverage in winter is for long time (still) different to summer conditions, we do not find that it is a decisive obstacle for energy production. Even though the Kara Sea (WSP2) is not ice free in the winter irrespective of the climate scenario, and also a significant part of the assessment unit off West Greenland is ice covered, in both cases the sea ice is thin enough to allow both for floating production units as well as LNG export. This is especially relevant as floating production units are the cheapest options for gas production in the Arctic (cf. Table 1). Differences in the analyzed climate scenarios do not lead to significant differences regarding the accessibility of the European Arctic Ocean for oil and gas production, at least not in the very likely spots studied here.

Next to bathymetry and sea ice conditions a number of additional factors impact on technology choice in the Arctic Ocean. The frequent presence of icebergs off the coast of West Greenland poses an immense risk to fixed platforms there. Contrary to floating platforms, fixed platforms cannot be uncoupled from the well and moved in the emergency that an approaching iceberg on collision course cannot be diverted by service vessels. The same environmental constraints occur to floating platforms, but due to the use of an emergency uncoupling feature the overall operating risk is lower for this family of platforms.

Regarding autonomous subsea production, the remoteness of the North Barents Sea is a significant hindrance. Subsea production is dependent on a floating storage and processing unit on the surface or, more commonly, on a receiving plant onshore. While a land base is theoretically thinkable on Franz Josef Land, in practice this is hardly feasible due to the remoteness and harsh environmental conditions on the island. We leave subsea production in combination with FPSU still in the technology portfolio, but operation in the North Barents Sea remains relatively unlikely.

## Discussion

Both floating and subsea systems are applicable in all assessment areas under study, even though the operation of subsea systems in the North Barents Sea remains unlikely. From Table 2 we know that production costs of natural gas amount to about 3.30 EUR per million British thermal unit (mmBtu) for floating systems and 4.05 EUR/mmBtu for subsea systems; for crude oil they are about 18.70 EUR/barrel for floating systems and 23.10 EUR/barrel for subsea systems. These numbers refer to a system with offshore processing, storage, and offloading, as we are focusing on offshore production, but processing, storage, and offloading onshore is not qualitatively different. All numbers were estimated in 2012. As is the case with any scenario analysis, our projections do only hold true under the assumptions posed by the global climate models. A similar caveat holds for the bottom-up, technology-driven cost estimates.

**Table 2:** Cost estimates for selected LNG and oil production technologies

Arctic LNG Option 1: FLNG (LNG FPSO)						
		1a: FLNG	1b: FLNG	1c: FLNG	1d: FLNG	1e: FLNG
		With onshore gas production	With Shallow water gas production	With floating gas production	With fixed platform gas production	With subsea gas production
Product rate ex receiving terminal	mtpa	3.5	3.5	3.5	3.5	3.5
Product rate ex receiving terminal (lifetime, 20 years)	mt	70.3	70.3	70.3	70.3	70.3
CAPEX						
Gas Production	Mio €	530	735	860	930	2820
FLNG Unit	Mio €	2510	2510	2510	2510	2510
LNG Carriers	Mio €	660	660	660	660	660
LNG Terminal	Mio €	450	450	450	450	450
Total CAPEX	Mio €	4150	4355	4480	4550	6440
OPEX						
Gas Production	Mio €/a	68	78	114	96	152
FLNG Unit	Mio €/a	150	150	150	150	150
LNG Carriers	Mio €/a	75	75	75	75	75
LNG Terminal	Mio €/a	38	38	38	38	38
Total OPEX	Mio €/a	331	341	377	359	415
Lifetime Cost (20 years)	Mio €	10 778	11 175	12 020	11 730	14 740
Specific Cost	€/MMBTU	2.96	3.07	3.30	3.22	4.05
Specific Cost	€/t LNG	153.4	159.0	171.0	166.9	209.7

Arctic LNG option 2: onshore LNG plant						
		2a: Onshore LNG	2b: Onshore LNG	2c: Onshore LNG	2d: Onshore LNG	2e: Onshore LNG
		With onshore gas production	With shallow water gas production	With floating gas production	With fixed platform gas production	With subsea gas production
Product rate ex receiving terminal	mtpa	3.5	3.5	3.5	3.5	3.5
Product rate ex receiving terminal (lifetime, 20 years)	mt	70.3	70.3	70.3	70.3	70.3
CAPEX						
Gas Production	Mio €	530	735	860	930	2820
Onshore LNG Unit	Mio €	2770	2770	2770	2770	2770
LNG Carriers	Mio €	660	660	660	660	660
LNG Terminal	Mio €	450	450	450	450	450
Total CAPEX	Mio €	4410	4615	4740	4810	6700
OPEX						
Gas Production	Mio €/a	68,4	78	114	96	152
FLNG Unit	Mio €/a	150	150	150	150	150
LNG Carriers	Mio €/a	75	75	75	75	75
LNG Terminal	Mio €/a	38	38	38	38	38
Total OPEX	Mio €/a	331.4	341	377	359	415
Lifetime Cost (20 years)	Mio €	11 038	11 435	12 280	11 990	15 000
Specific Cost	€/MMBTU	3.03	3.14	3.37	3.29	4.12
Specific Cost	€/t LNG	157.1	162.7	174.7	170.6	213.4

		Arctic Oil Option 1: Oil FPSO				
		1a: Oil FPSO	1b: Oil FPSO	1d: Oil FPSO	1c: Oil FPSO	1e: Oil FPSO
		With onshore oil production	With shallow water oil production	With floating oil production	With fixed platform oil production	With subsea oil production
Product rate ex receiving terminal	mtpa	2.7	2.7	2.7	2.7	2.7
Product rate ex receiving terminal (lifetime 20 years)	mt	54.9	54.9	54.9	54.9	54.9
CAPEX						
Oil Production	Mio €	530	735	760	930	2420
Oil FPSO Unit	Mio €	1320	1320	1320	1320	1320
Oil Tankers	Mio €	200	200	200	200	200
Oil Terminal	Mio €	160	160	160	160	160
Total CAPEX	Mio €	2210	2415	2440	2610	4100
OPEX						
Oil Production	Mio €/a	68	78	110	116	136
Oil FPSO Unit	Mio €/a	90	90	90	90	90
Oil Tankers	Mio €/a	50	50	50	50	50
Oil Terminal	Mio €/a	16	16	16	16	16
Total OPEX	Mio €/a	225	234	266	272	292
Lifetime Cost (20 years)	Mio €	6706	7103	7768	8058	9948
Specific Cost	€/MMBTU	2.36	2.50	2.73	2.83	3.50
Specific Cost	€/t Oil	122	129	142	147	181
Specific Cost	€/bbl Oil	15.5	16.5	18.0	18.7	23.1

Arctic Oil Option 2: Onshore Oil Plant						
		2a: Onshore Oil	2b: Onshore Oil	2d: Onshore Oil	2c: Onshore Oil	2e: Onshore Oil
		With onshore oil production	With shallow water oil production	With floating oil production	With fixed platform oil production	With subsea oil production
Product rate ex receiving terminal	mtpa	2.7	2.7	2.7	2.7	2.7
Product rate ex receiving terminal (lifetime 20 years)	mt	54.9	54.9	54.9	54.9	54.9
CAPEX						
Oil Production	Mio €	530	735	760	930	2420
Oil Onshore Unit	Mio €	1260	1260	1260	1260	1260
Oil Tankers	Mio €	200	200	200	200	200
Oil Terminal	Mio €	160	160	160	160	160
Total CAPEX	Mio €	2150	2355	2380	2550	4040
OPEX						
Oil Production	Mio €/a	68	78	110	116	136
Oil Onshore Unit	Mio €/a	80	80	80	80	80
Oil Tankers	Mio €/a	50	50	50	50	50
Oil Terminal	Mio €/a	16	16	16	16	16
Total OPEX	Mio €/a	214.8	224.4	256.4	262.4	282.4
Lifetime Cost (20 years)	Mio €	6446	6843	7508	7798	9688
Specific Cost	€/MMBTU	2.27	2.41	2.64	2.74	3.41
Specific Cost	€/t Oil	117	125	137	142	177
Specific Cost	€/bbl Oil	14.9	15.9	17.4	18.1	22.5

**Fig. 7:**
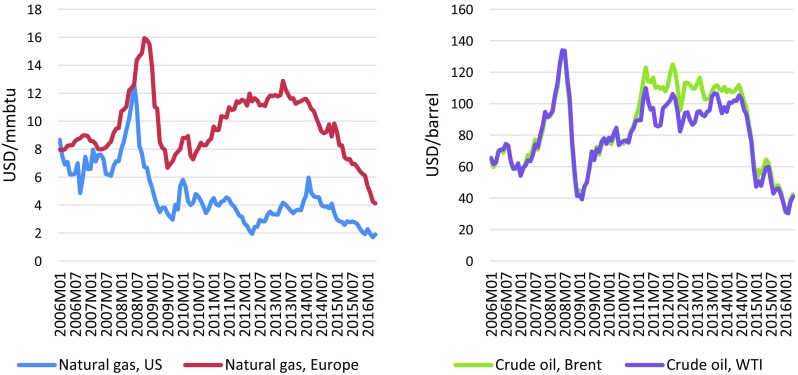
Gas and oil price developments on international commodity markets. Source: Own presentation based on World Bank (2016). Prices are in nominal US dollars per barrel (oil) or million British thermal unit (gas)

Figure 7 shows recent developments in the world market prices for oil and gas, with which Arctic offshore gas and oil would have to compete. Note that our cost estimates, which are below the current world market price for oil and at least the average gas price in Europe, do not take into account a number of relevant cost components. This includes internal interest requirements, risk premiums, political costs, e.g., for concession levies, or exploration and planning expenditures. These elements, however, might change the results to a certain degree, but not in general. Also, additional, location-dependent and highly uncertain costs for local infrastructure provision in the widely undeveloped Arctic may not have been fully taken into account in our estimations. Finally, while the reduction of sea ice might facilitate access to the Arctic Ocean, it will also likely have an impact on especially wave conditions in the once ice-covered areas, which may increase production costs. Under these circumstances and with market prices below or just above our estimated production costs we conclude that, based on our cost estimates, the large estimated quantities remain untapped as long as purely economic reasons determine the development decision.

At the same time we project that further sea ice reduction in the course of climate change will not be pivotal for offshore energy production in the European Arctic. Our analysis of a variety of global climate models indicates that, while in itself significant, the difference in sea ice conditions between the RCP 4.5 and RCP 8.5 scenarios even in 2040 is not decisive for the suitability or employment of the relevant production technologies: Scenario results suggest that in 2040, the ice has receded enough to make gas production technologically feasible in relevant areas even under RCP 4.5. This finding notwithstanding, we also show that climate change may of course have an impact north of the areas under study here. On economic grounds, recent oil and gas price developments, which give some indication of the upper limit of the highest marginal production cost in the market today, suggest that Arctic oil and gas will not be competitive in the near future. Also, in the light of global climate policies and protection goals, one might expect a decline in demand of fossil fuels questioning the rationality of exploiting Arctic oil and gas.

## Conclusions

Significant volumes of oil and natural gas resources are assumed in the Arctic Ocean. While their exploitation has not been feasible due to unfavorable climate and geographic conditions until today, global warming might improve accessibility in times to come. In this paper, we have shown that for parts of the European Arctic Seas, an exploitation of oil and gas might be technologically possible in the future. However, under current prices and with competing fossil and renewable energy sources, an exploitation does not seem to be rational from an economic point of view.

## Electronic supplementary material

Below is the link to the electronic supplementary material.

Supplementary material 1 (PDF 1,342 kb)

## Footnotes

1 Abbreviations correspond to USGS codes for the assessment areas.

2 EUMETSAT Ocean and Sea Ice Satellite Application Facility. Global sea ice concentration reprocessing dataset 1978-2015 (v1.2, 2015). Available from http://osisaf.met.no.

3 Centre de Recherche et d'Exploitation Satellitaire (CERSAT), at IFREMER, PlouzanĂŠ (France) covering 1991-present. Available from http://www.ifremer.fr.

4 Cost components considered in the cost estimation are included in Table S2.

## References

[CR1] Aarhus, B., A. Amundsen, R. Baustad, S.A. Eide, S. Erland, O. Nestaas, Ø. Rossebø, N. Rustad, et al. 2014. Barents sea gas infrastructure. DMS Document Number 99807, Gassco.

[CR2] Bambulyak A, Frantzen B (2005). Oil transport from the Russian part of the Barents Region; Status per January 2005.

[CR3] Casey, K. 2014. Greenland’s new frontier: Oil and gas licences issued, though development likely years off. The Arctic Institute—Center for Circumpolar Security Studies. Retrieved 22 May, 2016, http://www.thearcticinstitute.org/2014/01/greenlands-new-frontier-oil-and-gas.html.

[CR5] Emmerson, C., and G. Lahn. 2012. Arctic opening: Opportunity and risk in the high north. Chatham House, Lloyd’s.

[CR6] Gautier DL, Bird KJ, Charpentier RR, Grantz A, Houseknecht DW, Klett TR, Moore TE, Pittman JK (2009). Assessment of undiscovered oil and gas in the Arctic. Science.

[CR7] Harsem O, Heen K, Rodrigues JMP, Vassdal T (2015). Oil exploration and sea ice projections in the Arctic. Polar Record.

[CR8] IBRU. 2012. Maritime Jurisdiction and boundaries in the Arctic Region. IBRU: The Centre for Borders Research at Durham University, http://www.durham.ac.uk/ibru/resources/arctic.

[CR9] International Energy Agency (IEA). 2013. Resources to Reserves. IEA/OECD, Paris. (Chapter 4: Trends and challenges of frontier oil and gas, p. 135.: Technologies for meeting the Arctic challenge).

[CR10] Lindholt L, Glomsrod S (2012). The Arctic: No big bonanza for the global petroleum industry. Energy Economics.

[CR11] Moss RH, Edmonds JA, Hibbard KA, Manning MR, Rose SK, van Vuuren DP, Carter TR, Emori S (2010). The next generation of scenarios for climate change research and assessment. Nature.

[CR15] Overland I, Bambulyak A, Bourmistrov A, Gudmestad O, Mellemvik, Zolotukhin A, Bourmistrov A, Mellemvik F, Bambulyak A, Gudmestad O, Overland I, Zolotukhin A (2015). Barents Sea oil and gas 2025—Three scenarios. International Arctic Petroleum Cooperation: Barents Sea Scenarios.

[CR16] Riemann-Campe, K., M. Karcher, F. Kauker, and R. Gerdes. 2014. D1.51 Results of Arctic ocean-sea ice downscaling runs validated and documented. Project deliverable report. http://www.access-eu.org/modules/resources/download/access/Deliverables/D1-51-AWI-final.pdf.

[CR17] Taylor KE, Stouffer RJ, Meehl GA (2012). An Overview of CMIP5 and the Experiment Design. Bulletin of the American Meteorological Society.

[CR22] USGS. 2008a. Circum-Arctic Resource Appraisal: Estimates of Undiscovered Oil and Gas North of the Arctic Circle. USGS Fact Sheet 2008-3049.

[CR23] USGS. 2008b. Summary Statistics of Results from the Circum-Arctic Resource Appraisal: Assessment Unit Codes correspond to labels on AUs shown in Figures 1 and 2.

[CR24] World Bank. 2016. World Bank Commodity Price Data (The Pink Sheet). Retrieved Nov 22, 2001, from http://www.worldbank.org/en/research/commodity-markets.

