# Supplementary material for: Climate change, future Arctic Sea ice, and the competitiveness of European Arctic offshore oil and gas production on world markets
Source: Ambio. 2017 Oct 24;46(Suppl 3):410–22. doi: 10.1007/s13280-017-0957-z (PMC5673873; doi:10.1007/s13280-017-0957-z)
Supplement: Supplementary file 1 — Supplementary material 1 (PDF 1,342 kb) [file 13280_2017_957_MOESM1_ESM.pdf]

Electronic Supplementary Material

Title: Climate change, future Arctic Sea ice, and the competitiveness of European Arctic offshore oil and gas production on world markets

Authors: Sebastian Petrick, Kathrin Riemann-Campe, Sven Hoog, Christian Growitsch, Hannah Schwind, Rüdiger Gerdes, Katrin Rehdanz

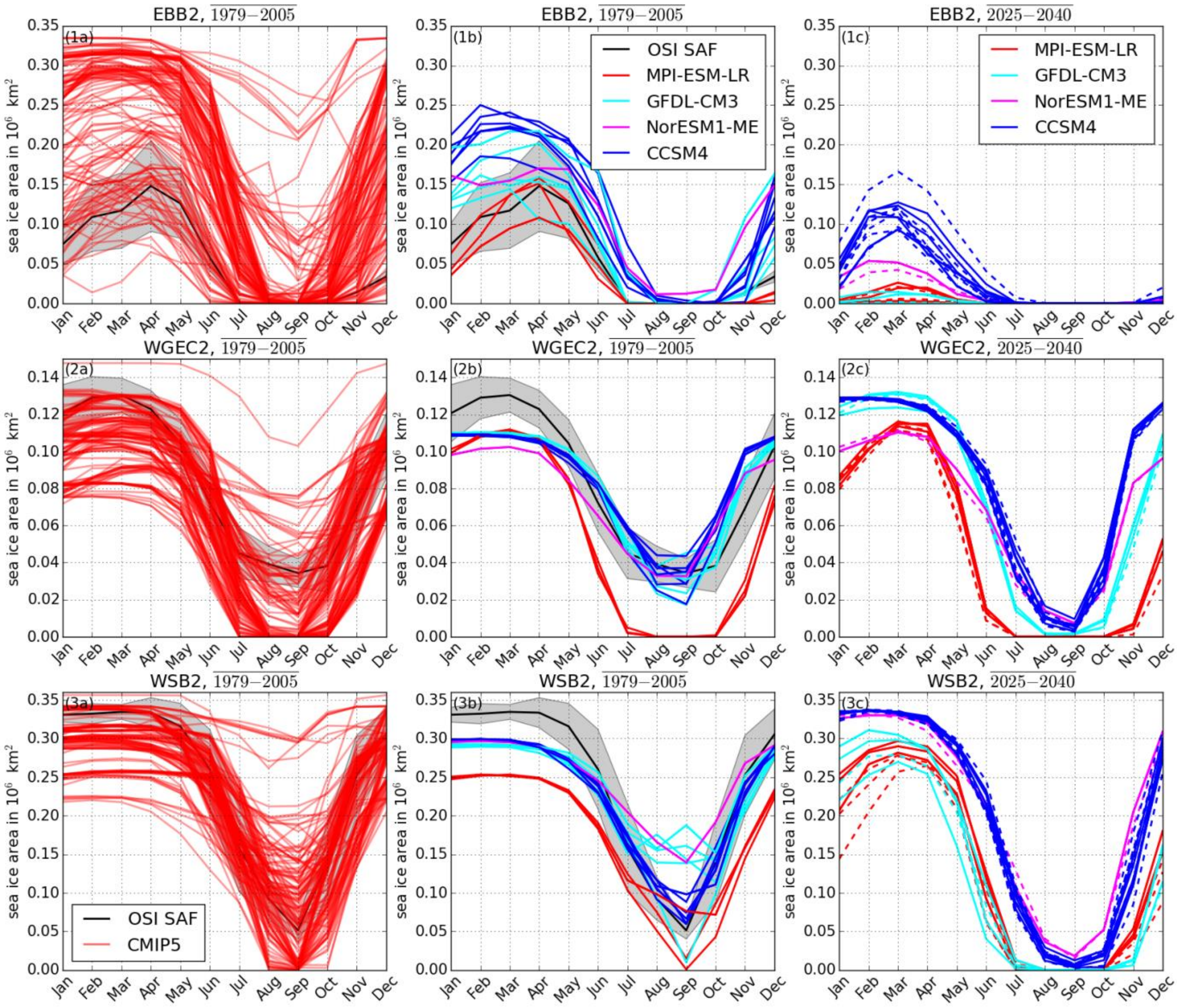

Fig. S1 Mean seasonal cycle of sea ice area in million km^2 for regions (1) EBB2 in the southern Barents Sea, (2), WGEC2 off the west coast of Greenland and (3) WSB2 in the Kara Sea: (a) Mean over the years 1979-2005 of satellite derived data OSI SAF (mean: solid line, standard deviation:grey shading) and single ensemble members of CMIP5 models; historical simulation. (b) Mean over the years 1979-2005 of satellite derived data OSI SAF (mean: solid line, standard deviation:grey shading) and four selected CMIP5 models; historical simulation. (c) Mean over the time period 2025-2040 from for CMIP5 models with emission scenarios RCP 4.5 (solid lines) and RCP 8.5 (dashed lines).

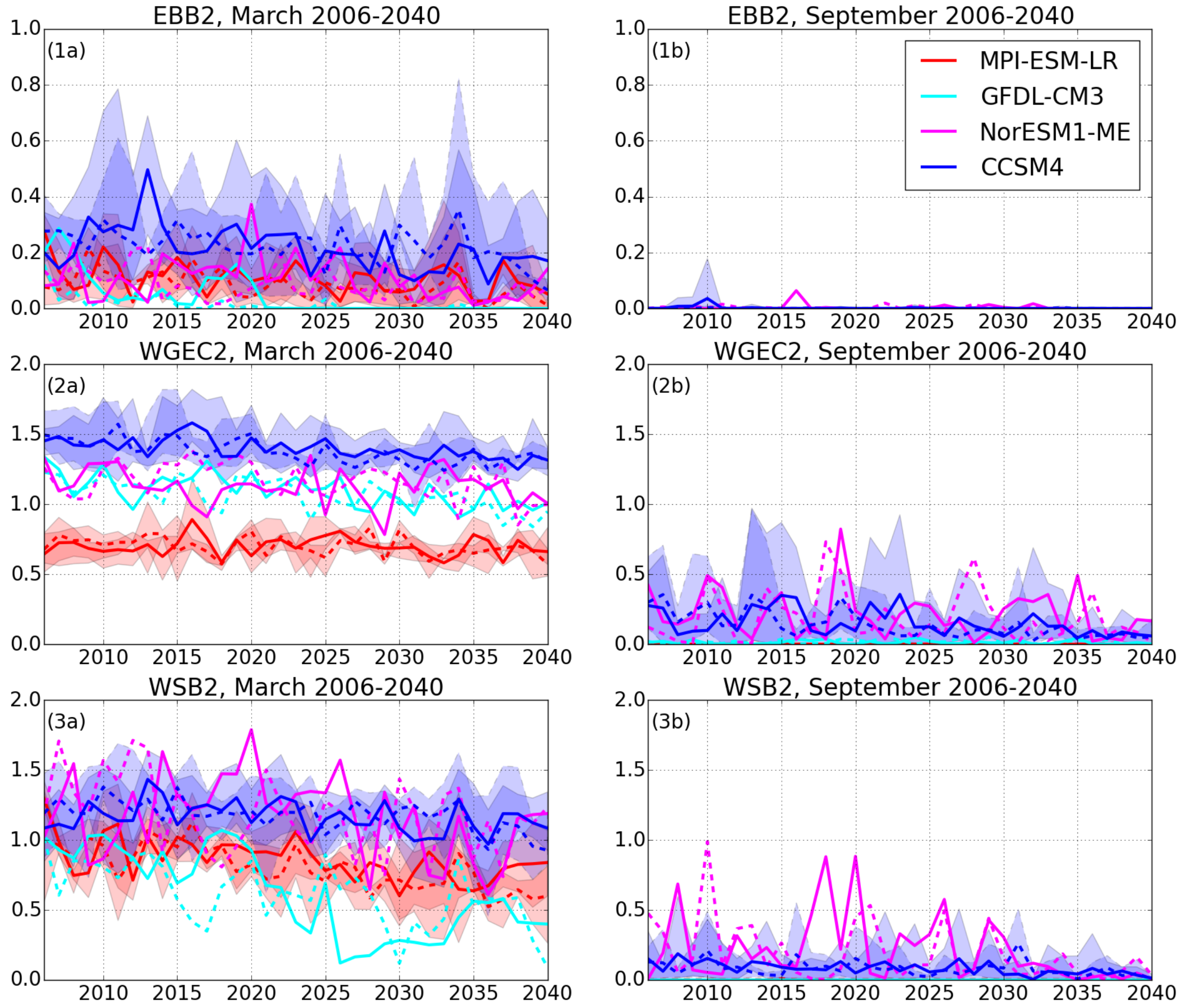

Fig. S2 March (a) and September (b) mean sea ice thickness in m for the regions (1) EBB2 in the southern Barents Sea, (2), WGEC2 off the west coast of Greenland and (3) WSB2 in the Kara Sea from four CMIP5 models with the emission scenarios RCP 4.5 (solid lines) and RCP 8.5 (dashed lines). If more than one ensemble member is available per model, the mean is shown by the line and the range over all ensemble members per model is indicated by the shading.

Table S1 List of CMIP5 models analysed including modeling groups and their terms of use:

Modeling Center (or Group)	Institute ID	Model Name
	CSIRO-BOM	ACCESS1.0
Beijing Climate Center, China Meteorological Administration	BCC	BCC-CSM1.1
Canadian Centre for Climate Modelling and Analysis	CCCMA	CanESM2<br>CanCM4
National Center for Atmospheric Research	NCAR	CCSM4
Community Earth System Model Contributors	NSF-DOE-NCAR	CESM1(CAM5.1,FV2)
Centre National de Recherches Météorologiques / Centre Européen de Recherche et Formation Avancée en Calcul Scientifique	CNRM-CERFACS	CNRM-CM5
Commonwealth Scientific and Industrial Research Organization in collaboration with Queensland Climate Change Centre of Excellence	CSIRO-QCCCE	CSIRO-Mk3.6.0
EC-EARTH consortium	EC-EARTH	EC-EARTH
LASG, Institute of Atmospheric Physics, Chinese Academy of Sciences and CESS,Tsinghua University	LASG-CESS	FGOALS-g2
LASG, Institute of Atmospheric Physics, Chinese Academy of Sciences	LASG-IAP	FGOALS-s2
NOAA Geophysical Fluid Dynamics Laboratory	NOAA GFDL	GFDL-CM3<br>GFDL-ESM2G<br>GFDL-ESM2M
NASA Goddard Institute for Space Studies	NASA GISS	GISS-E2-H<br>GISS-E2-R
National Institute of Meteorological Research/Korea Meteorological Administration	NIMR/KMA	HadGEM2-AO
Met Office Hadley Centre (additional HadGEM2-ES realizations contributed by Instituto Nacional de Pesquisas Espaciais)	MOHC (additional realizations by INPE)	HadCM3<br>HadGEM2-CC<br>HadGEM2-ES
Institute for Numerical Mathematics	INM	INM-CM4
Institut Pierre-Simon Laplace	IPSL	IPSL-CM5A-LR<br>IPSL-CM5A-MR<br>IPSL-CM5B-LR

Japan Agency for Marine-Earth Science and Technology, Atmosphere and Ocean Research Institute (The University of Tokyo), and National Institute for Environmental Studies	MIROC	MIROC-ESM<br>MIROC-ESM-CHEM
Atmosphere and Ocean Research Institute (The University of Tokyo), National Institute for Environmental Studies, and Japan Agency for Marine-Earth Science and Technology	MIROC	MIROC4h<br>MIROC5
Max-Planck-Institut für Meteorologie (Max Planck Institute for Meteorology)	MPI-M	MPI-ESM-MR<br>MPI-ESM-LR<br>MPI-ESM-P
Meteorological Research Institute	MRI	MRI-CGCM3
Norwegian Climate Centre	NCC	NorESM1-M<br>NorESM1-ME

Output from yellow highlighted models is available for unrestricted use. Output from the others may only be used for non-commercial research and educational purposes. [See complete "Terms of Use": http://cmip-pcmdi.llnl.gov/cmip5/terms.html].

Table S2 Cost components considered in the cost estimation.

Production / processing technology	Production supporting technology	Product transmission and export technology
• Floating production facilities (FPSO / FLNG)<br>• Subsea production facilities<br>• Fixed (concrete) production platforms<br>• Fixed shallow water production facilities<br>• Onshore production facilities<br>• Onshore plants for<br>• raw product receiving<br>• treatment (acid gas removal and drying)<br>• liquefaction or compression or GTL processing<br>• storage of products<br>• service station for offshore facilities	• Drilling vessels (for appraisal wells and workover)<br>• Offshore supply vessels (with ice breaking ability)<br>• Helicopters<br>• Sea going emergency, evacuation and rescue (EER) systems	• Riser (oil, gas)<br>• Pipelines (oil, gas)<br>• Flexible pipes and hard arms (for oil, gas or liquefied gas) for loading<br>• Turrets, mooring systems and shuttle tankers (for gas or liquefied gas or oil)

Floating Production Facility

Floating production facilities can be both, platforms with a single main functionality (production and send-out of hydrocarbons) and platforms with combined functionality, production, treatment, processing and send-out.

All vessels operating in the Arctic have to provide ice breaking abilities and suitable rescue and evacuation schemes. Floating production facilities are permanently moored at location by means of internal turret mooring systems with gas production abilities.

In general, a cost share of 20-30 % of the overall costs has to be considered for oil / gas production.

Subsea Production Facility

Production from totally submerged facilities has the main advantage that during normal production no surface piercing structure has to withstand harsh environments and loads from drifting ice or icebergs. Nevertheless, the wells have to be maintained from time to time or in an emergency by means of work over drilling. Drilling vessels with significant ice breaking abilities have to be employed. These vessels are rare and expensive so that they are not purchased but long term contracted for the complete production campaign of >20 years.

The produced multiphase flow of gas, oil, water, and sand is processed and exported via pipeline to the shore receiving plant, where also the local operators of the subsea system are accommodated.

In general, a cost share of 20-30 % of the overall costs has to be considered for oil / gas production with transport from the reservoir to the treatment plant, including control umbilical and export pipelines.

Fixed Production Facility

The fixed concrete platform option provides production, pre-treatment and storage capabilities. These platforms are restricted in (deck) space and are normally not suited to allow liquefaction of gas. Thus, the produced gas is sent to a shore liquefaction plant via pipeline. Produced oil and condensate can be either exported via scheduled shuttle tankers or pumped to shore via pipeline.

As the platform cannot be moved from location, it has to withstand all occurring environmental conditions like severe sea states, heavy winds and icing of deck structures as well as drifting ice or icebergs. Consequently, ice management by means of ice breaking Offshore Supply Vessels (OSVs) has been considered to reduce these loads to the fixed structure.

In general, a cost share of 20-30 % of the overall costs has to be considered for oil / gas production with transport from the reservoir to the treatment plant, including gas processing and associated pipelines.

Shallow Water Production Facility

As stated above production facilities can be installed in shallow or even very shallow water. Examples are available for facilities which are constructed in benign areas like in middle or south of Europe and towed to an Arctic location by means of tugs.

Installation takes place at a prepared berm e.g. made of gravel. Due to a relatively short distance to shore and possibly restricted water depth send-out of the products is carried out by pipelines to separate onshore treatment and export facilities. The shallow water facilities have to be protected against drifting ice by means of passive ice barriers, optionally aided by an active ice management with Offshore Supply Vessels (OSVs).

In general, a cost share of 15-25 % of the overall costs has to be considered for oil / gas production with transport from the reservoir to the treatment plant, including gas processing and associated pipelines.

Onshore Production Facility

Production facilities located onshore in the Arctic have to consider related harsh environmental constraints. Although no wave, ice or iceberg loads can occur it is possible that future climatic changes might result in melting surface soils or even the development of swamp areas.

Due to the demanding environments in the Arctic construction at location often suffers from significant time delays caused by insufficient infrastructure, complicate or unsteady material supply, or problems with personnel provision. Nevertheless, realized examples can be found for Alaska and Siberia.

In general, a cost share of 15-20 % of the overall costs has to be considered for oil / gas production with transport from the reservoir to the treatment plant, including gas processing and associated pipelines.

Treatment, Storage, Loading, Shipping and receiving for gas production
Treatment of the raw products gas and oil is required to allow either send-out to the connected gas network or to export tankers. Case dependent treatment can comprise de-hydration, de-sanding, recovery of flow assuring inhibitors (e.g. Methyl Ethylene Glycol, MEG) and removal of CO_2, mercury or N_2 and the liquefaction of the gas for efficient storage and transportation. Liquids like LNG, CNG or GTL products are stored until they are loaded to dedicated shuttle tankers. These tankers provide the worldwide market with the required energy deliveries, which can be comparably handled in terms of quantities of British Thermal Units (BTU).

For the assessment a mean transportation distance between the treatment plant and the European receiving plant of 5000 km has been considered.

Treatment, Storage, Loading, Shipping and receiving for oil production
Treatment of the raw oil is required to allow either send-out to the connected pipeline network or to export tankers. Case dependent treatment can comprise de-hydration, de-sanding, recovery of flow assuring inhibitors (e.g. MEG) and removal of CO_2, mercury or N_2 for efficient storage and transportation. Especially transportation by tankers allows providing the worldwide market with the required energy deliveries, which can be comparably handled in terms of quantities of British Thermal Units (BTU). Again a mean transportation distance between the treatment plant and the European receiving plant of 5000 km has been considered.

Table S3 Data and information sources for cost estimates.

Source	Information contained
www.offshore-technology.com; www.upstreamonline.com	Online databases providing general information about license holders, operators, technology, investments of international oil and gas projects.
Norwegian Petroleum Directorate: http://factpages.npd.no/factpages/	Database providing general information about license holders, operators, technology, investments of Norwegian oil and gas projects.
The Economy 2010, www.economics.gov.nl.ca	Provides information about oil and gas production economic performance in Newfoundland and Labrador and medium term outlook.
Energy Information Agency (EIA), Russia, Country Analysis Briefs, www.eia.doe.gov, Nov. 2010	General information about energy production, consumption, stakeholder, (Oil and Gas) investments, outlook.
EIA, Norway, Country Analysis Briefs, www.eia.doe.gov, Aug. 2010	Compare EIA (Russia).
EIA, Canada, Country Analysis Briefs, www.eia.doe.gov, July 2009	Compare EIA (Russia).
EIA: *The Global Liquefied Natural Gas Market: Status and Outlook*, www.eia.gov/oiaf/analysispaper/global/lngindustry.html, December 2003;	Information about costs for gas production, liquefaction, shipping, storage, regasification.
Bambulyak, A., and B. Frantzen. 2005. Oil transport from the Russian part of the Barents Region; Status per January 2005. Kirkenes: Norwegian Barents Secretariat.	Information about oil and gas production, infrastructure, transport volumes, logistics along Northern Sea Route.
Chan, L., G. Eynon, and D. McColl. 2005. The Economics of High Arctic Gas Development: Expanded Sensitivity Analysis. Calgary: Canadian Energy Research Institute.	Comparison of most relevant gas production and export scenarios including relevant cost contributors.
OPEC. 2010. World Oil Outlook, OPEC Secretariat, Vienna, Austria	Status quo of oil and gas production, demand, world economic development, outlook to 2030, consideration of all relevant fields of the energy supply chain.
